# The role of methylation profiling in histologically diagnosed neurocytoma: a case series

**DOI:** 10.1007/s11060-022-04117-1

**Published:** 2022-08-22

**Authors:** Adam Z. Kalawi, Denise M. Malicki, Zied Abdullaev, Drew W. Pratt, Martha Quezado, Kenneth Aldape, Jennifer D. Elster, Megan R. Paul, Paritosh C. Khanna, Michael L. Levy, John R. Crawford

**Affiliations:** 1 Division of Child Neurology, Department of Neurosciences, University of California, San Diego, CA USA; 2 Rady Children’s Hospital, San Diego, CA USA; 3 Division of Hematology Oncology, Department of Pediatrics, University of California, San Diego, CA USA; 4 Department of Radiology, University of California, San Diego, CA USA; 5 Department of Pathology, University of California, San Diego, CA USA; 6 Division of Pediatric Neurosurgery, Department of Neurosurgery, University of California, San Diego, CA USA; 7 Laboratory of Pathology, National Cancer Institute, Bethesda, MD USA; 8 Department of Pathology, University of Michigan, Ann Arbor, MI USA; 9 Division of Child Neurology, Children’s Hospital of Orange County, Orange, CA USA

**Keywords:** Pediatric brain tumor, Pediatric neurocytoma, Neurocytoma, Atypical neurocytoma, Methylation

## Abstract

**Purpose:**

To highlight the clinical, neuroradiographic, neuropathologic, and molecular features of histologically identified neurocytoma in a pediatric cohort and highlight the evolving use methylation profiling in providing diagnostic clarity in difficult to diagnosis pediatric brain tumors.

**Methods:**

Five consecutive children (ages 9–13, 2 girls 3 boys) were histologically diagnosed with neurocytoma at Rady Children’s Hospital San Diego from 2012 to 2018. Clinical and molecular features were analyzed with regards to treatment course and outcome.

**Results:**

Presenting symptoms included seizures (n = 2), syncope (n = 1), headache (n = 2), visual disturbances (n = 2) and emesis (n = 2). Tumor location included intraventricular (n = 2), intraventricular with parenchymal spread (n = 1), and extraventricular (n = 2). Magnetic resonance imaging demonstrated reduced diffusivity (2/5), signal abnormality on susceptibility-weighted sequences (3/5), and varying degrees of contrast enhancement (4/5). All patients underwent surgical resection alone. Recurrence occurred in four children that were treated with surgery (4/4), adjuvant radiation (2/4), and chemoradiation (1/4). Neuropathologic features included positivity for GFAP (4/5), synaptophysin (4/5), NSE (2/2), NeuN (4/4), and variable Ki-67 (< 1% to 15%). Next generation sequencing (3/5) and microarray (3/5) collectively were abnormal in four of five tumors. Methylation profiling was successfully performed on four of five samples which led to modification of diagnosis in two patients and the others were either unclassifiable or confirmatory with the histologic diagnosis. Mean time to follow up was 77 months (range 44–112 months). Mean progression free survival and overall survival were 24 months (range 6 to 52 months) and 100% respectively.

**Conclusion:**

Neurocytomas are a rare clinical entity that warrants further investigation into molecular and pathologic prognosticating features. Methylation profiling may aid in differentiation of neurocytoma from other difficult to diagnose tumors who share similar histologic features.

## Introduction

Central and extraventricular neurocytomas are rare central nervous system tumors of neuronal origin recognized by the fifth edition of World Health Organization (WHO) classification of tumors of the central nervous system that were first described histologically in 1982[1, 2]. These tumors account for only 0.1–0.5% of all brain tumors with a reported overall annual incidence of 0.032 per 100,000 population, initial presentation age ranging from the prenatal period to 81 years of age, and peak age of diagnosis at 20–34 years of age [3–6]. Neurocytomas are subdivided into central neurocytomas (CNs), located within the ventricular system, and extra-ventricular neurocytomas (EVNs) owing to their similar histologic characteristics but variable regional occurrence and clinical presentations [7]. Though the majority of these tumors have benign characteristics with excellent response to surgical intervention, a subset of neurocytomas have a predilection for recurrence or dissemination and have been described as “atypical neurocytomas”(ANs) [8, 9]. The precise definition of ANs is not agreed upon in the literature or latest edition of the WHO classification of central nervous system tumors, however there is a consensus that MIB-1 staining for Ki-67 antigen with values greater than 2–3%, with or without cellular atypia, have significant association with a worse prognosis [8, 10–12]. Though surgical intervention with goal of gross total resection is often the first step in the management of these brain tumors, there is no definitive consensus on the management of incompletely resected, atypical, or recurrent tumors with respect to surgical, radiation, or chemotherapeutic interventions [13–17].

The management of neurocytomas in the pediatric population represents a unique challenge as the body of available literature is based primarily on the management of neurocytomas in young adults, with the pediatric literature being limited to case series and small sample size reviews [18, 19]. Furthermore, little is known about the molecular drivers of neurocytomas which limits the potential for tailored therapies in the era of personalized medicine. As the field of neuro-oncology moves towards the adoption of next-generation sequencing (NGS) and methylation profiling, rare CNS tumors such as neurocytomas may be early beneficiaries of this targeted diagnostic and treatment strategy [20, 21]. We present five unusual cases of histologically diagnosed neurocytomas with correlated clinical, radiographic, pathologic, and molecular characteristics. Our results highlight the clinical utility of methylation profiling in distinguishing neurocytoma from other difficult to diagnose pediatric brain tumors with similar histologic characteristics, adding to the molecular diagnostic complexity of this rare pediatric central nervous system tumor.

## Materials and methods

A retrospective chart review was performed at Rady Children’s Hospital San Diego from 2012 to 2018 and identified five consecutive children (age at diagnosis 9–13, 2 girls 3 boys) that were histologically diagnosed with neurocytoma. A comprehensive chart review was performed through January 1, 2022, which provided key statistical, narrative, and demographic elements of each case. Surgical pathology was reviewed by a neuropathologist. Intervals for neurologic imaging were decided clinically by the treating neuro-oncology team. Radiographic findings were reviewed by a pediatric neuroradiologist as well as the treating neuro-oncology team. All samples were formalin-fixed and paraffin-embedded. NGS and chromosomal microarray were performed on all samples. Methylation analysis was performed by the laboratory of pathology at the National Cancer Institute, in which tumor samples were evaluated for content (minimum 50% cutoff) and areas of high cellularity underwent DNA extraction followed by bisulfite conversion (EZ DNA Methylation Kit, Zymo Research D5001), further processing with the Infinium FFPE DNA Restore kit (Illumina, USA), and subsequent assay on the Infinium MethylationEPIC 850 K kit (Illumina, USA). All beadchips were scanned on iScan reader and output idat files were processed through CNS classifier versions v11b6 [21] and v12 (publicly available, unpublished) of the CNS tumor methylation classifier [21]. Classification calibrated cutoff scores of > 0.85 were used unless otherwise specified, denoting a high confidence classification. This study was approved by the institutional review board (IRB) at the University of California, San Diego.

## Results

The demographic, clinical, radiographic, and histologic features of the neurocytoma series are summarized in Table 1. Five consecutive patients were identified to have a histologic diagnosis of neurocytoma, 2 girls and 3 boys with a mean age at time of initial imaging identification of lesion of 8 (range 2–13), and mean age at time of histologic diagnosis of 11 (range 9–13). Presenting symptoms included seizures (n = 2), syncope (n = 1), headache (n = 2), visual disturbances (n = 2) and emesis (n = 2). Tumor location varied, including intraventricular (n = 2), intraventricular with parenchymal spread (n = 1), and extraventricular (n = 2). The two patients who presented with seizures (cases 4 and 5) were found to have extraventricular tumors, with long intervals between initial lesion identification and tumor resection due to radiographic stability on initial imaging.

**Table 1:** Demographic, Clinical, Radiographic, and Histology Characteristics

Patient #	Demographics, Presentation, and Location	Recurrences and Progression Free Survival	Treatments	Tumor Size	DWI	SWI	GAD	GFAP	Ki-67	Synaptophysin	NSE	NeuN
1	12 y/o female with weakness, nausea vomiting, diplopia, and papilledema, identified to have mass within the lateral ventricles with associated hydrocephalus	1 recurrence after 10 months progression free survival	Two surgical interventions, two adjuvant radiation therapies	4.1 × 3.2 × 4.3 cm	+	+	+ +	–	10–15%	+	+	+
2	13 y/o male with headache, nausea, vomiting, and hemianopsia, identified to have right lateral ventricle mass with parietal parenchymal spread	5 recurrences after 6 months progression free survival	Three surgical interventions, two adjuvant radiation therapies, two chemotherapeutic interventions	7.1 × 5.6 × 6.7 cm	+	+	+ +	+	2–12%	–	+	+
3	11 y/o female with syncopal episode, identified to have a right lateral ventricle mass	0 recurrences with 44 months of progression free survival to date	One surgical intervention	0.9 × 1.1 × 1.1 cm	–	–	–	+ + +	< 1%	+	N/A	+
4	11 y/o male with focal epilepsy identified to have right temporoparietal mass	2 recurrences with 10 months of progression free survival	Two surgical interventions with one short-interval revision	3.8 × 1.9 × 2.7 cm	–	–	+	+ + +	< 1%	+	N/A	+
5	9 y/o male with focal epilepsy identified to have left parietal lobe mass	1 recurrence with 52 months of progression free survival	Two surgical interventions	4.0 × 3.5 × 3.3 cm	–	+	+	+ + +	< 1%	+	N/A	N/A

*DWI* *Diffusion-weighted Imaging, SWI* *Susceptibility-weighted imaging, GAD* *Gadolinium enhancement, GFAP* *Glial fibrillary acidic protein, NSE* *Neuron specific enolase, NeuN* *Neuronal nuclei,* + *Minimal,* +  + *Moderate,* +  +  + *Avid*

Two patients (cases 2 and 4) had multiple disease recurrences with unique clinical courses. Case 2 had a prolonged clinical course with 6 months of progression free survival after initial sub-total resection, followed by courses of x-ray radiation therapy, bevacizumab, additional sub-total resection, trametinib, and most recently proton therapy. Case 4 had a history of cardiac transplantation and initially was managed for focal epilepsy thought to be due to a focal cortical dysplasia. Though initially stable on MRI, the patient’s lesion demonstrated growth on subsequent imaging and was later histologically identified as an extraventricular neurocytoma. This tumor was initially managed with surgical resection and short-interval revisions, with 10 months of progression free survival thereafter.

**Fig. 1:**
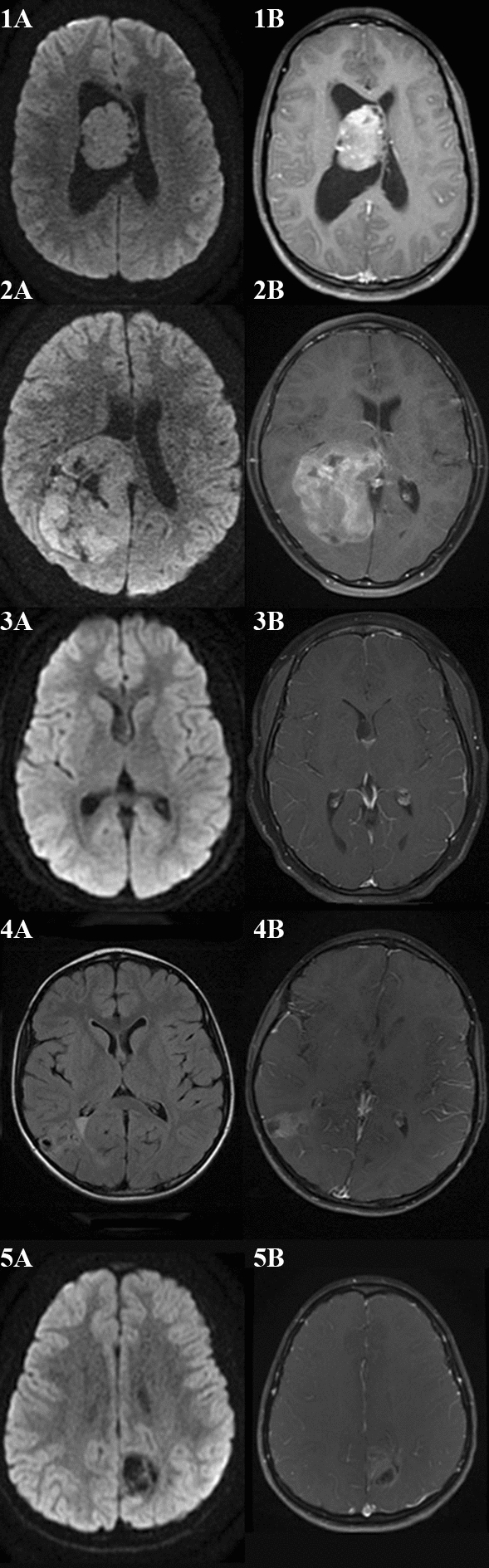
MRI of patient 1 demonstrates a central intraventricular tumor with reduced diffusivity (1A) and avid enhancement on post-gadolinium T1-weighted sequences (1B). MRI of patient 2 demonstrates a large posterior right lateral ventricle tumor with parietal and hemispheric parenchymal spread with reduced diffusivity (2A) and avid heterogenous contrast enhancement on post-gadolinium T1-weighted sequences (2B). MRI of patient 3 demonstrates a subtle intraventricular tumor adjacent to the septum pellucidum and right lateral ventricle on diffusion weighted sequences (3A) with minimal contrast enhancement likely reflecting choroid inferiorly on post-gadolinium T1-weighted sequences (3B). MRI of patient 4 demonstrates a right posterior temporoparietal mixed solid and cystic tumor on T2-weight fluid attenuated inversion recovery sequences (4A) with moderate enhancement on post-gadolinium T1-weighted sequences (4B). MRI of patient 5 demonstrates a mixed solid and cystic left posterior frontoparietal tumor without restricted diffusion on diffusion weighted sequences (5A) and moderate enhancement on post-gadolinium T1-weight sequences (5B).

Initial MRIs demonstrated tumors with reduced diffusivity (2/5), punctate areas of signal abnormality on susceptibility-weighted sequences (3/5), and varying degrees of contrast enhancement (4/5) (Fig. 1). One patient presenting with seizures had an initial neuroimaging appearance most consistent with a focal cortical dysplasia prior to subsequent progression. All patients were negative for leptomeningeal and spinal metastatic disease on initial and subsequent neuroimaging.

**Fig. 2:**
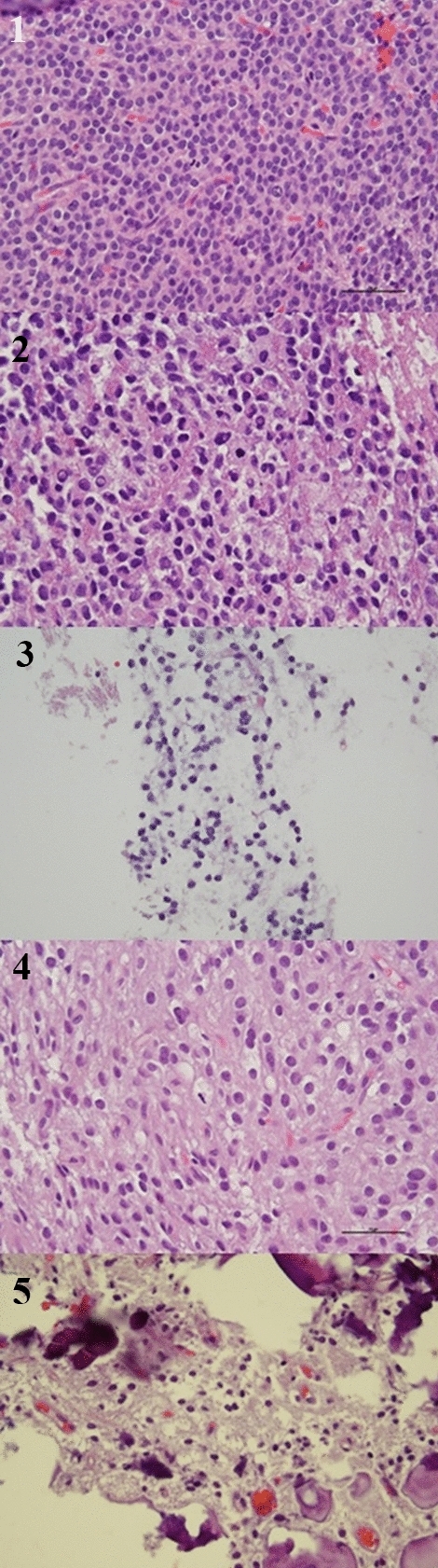
Sample from patient 1 demonstrates highly cellular proliferation of uniform cells with round nuclei, stippled chromatin, moderately eosinophilic cytoplasm, and mitotic activity with 6 mitotic figures in 10 high-power fields. Sample from patient 2 demonstrates moderately cellular proliferation of polygonal cells with moderate nuclear pleomorphism, stippled chromatin, mitotic activity with 8 mitotic figures in 10 high-power fields, and necrosis. Sample from patient 3 demonstrates moderately cellular proliferation with round, regular nuclei, and stippled chromatin. Sample from patient 4 demonstrates moderately cellular proliferation with round nuclei showing perinuclear “halos” transitioning into more mature neuronal forms, extensive calcification without eosinophilic granular bodies or perivascular lymphocytic cuffing. Sample from patient 5 demonstrates moderately cellular proliferation with uniformly round nuclei, perinuclear “halo” formation, and extensive calcification.

Neuropathologic features included positivity for GFAP (4/5), synaptophysin (4/5), NSE (2/2), NeuN (4/4), and variable Ki-67 (< 1% to 15%). Two patients had Ki-67 staining > 2–3% and would thus be histologically described as atypical neurocytomas (Fig. 2).

The molecular features of this cohort are summarized in Table 2. NGS (3/5) and microarray (3/5) collectively were abnormal in 4 of 5 tumors. Case 1 demonstrated gains in chromosome 5 alone, whereas cases 4 and 5 demonstrated more broad chromosomal gains on copy number analysis (Table 2). NGS abnormalities included an *EWSR-ATF1* gene fusion and a *MUTYH* mutation in case 2, seven gene variants of uncertain significance (VUS) in case 1, and a single VUS in the ATM gene in case 5.

**Table 2:** Molecular Characteristics

Patient #	Next generation sequencing	Chromosomal microarray	Methylation profiling	Histologic diagnosis	Integrated diagnosis
1	Seven variants of uncertain significance	Gains in chromosome 5	Central neurocytoma, Calibrated score: 0.999	Central atypical neurocytoma	Central atypical neurocytoma
2	*EWSR-ATF1* gene fusion *MUTYH* mutation	No significant findings	No match	Central atypical neurocytoma with *EWSR-ATF1* gene fusion and *MUTYH* mutation	Neuroepithelial neoplasm with *EWSR1-ATF* gene fusion and *MUTYH* mutation
3	No clinically significant findings	No significant findings	Inadequate sample given cystic nature of tumor	Central neurocytoma	Central Neurocytoma
4	No clinically significant findings	Gains in chromosomes 5, 7, 11, 12, 19, 20, X, and Y	Ganglioglioma, calibrated score: 0.927	Extraventricular neurocytoma	Altered diagnosis to ganglioglioma
5	One variant of uncertain significance in the *ATM* gene	Gains in chromosomes 6, 7, 8, X, Y	Ganglioglioma, calibrated score: 0.766	Extraventricular neurocytoma	Altered diagnosis to ganglioglioma

**Fig. 3:**
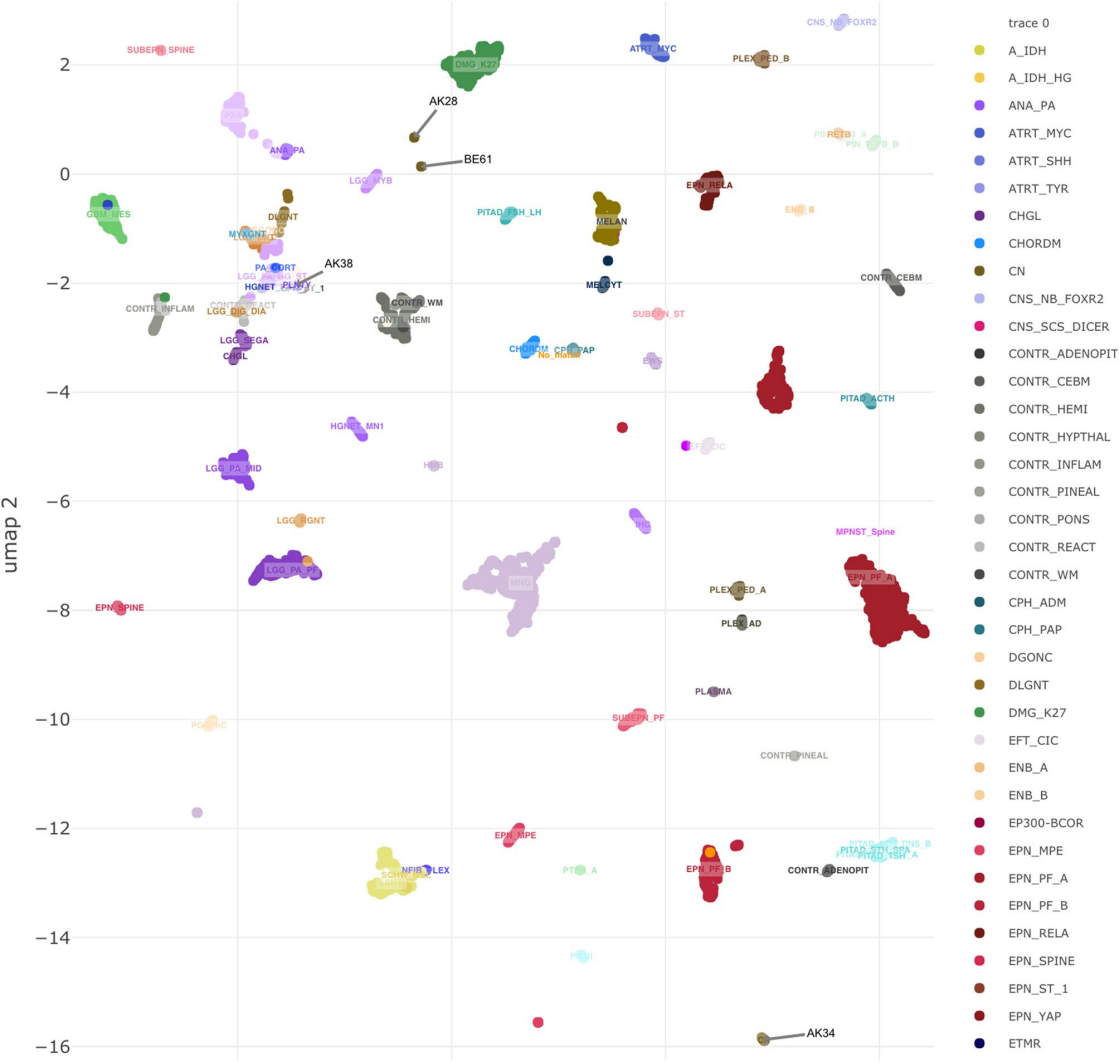
Cluster analysis of the four patients with successful methylation testing are shown. Patient 1 (AK34) clustered with central neurocytoma (calibrated score=0.999). Patient 2 (AK28) did not cluster with any known entity. Patient 4 (AK38) clustered with ganglioglioma (calibrated score 0.927). Patient 5 clustered most closely to a ganglioglioma (calibrated score 0.766).

Methylation profiling was attempted on all samples to provide an integrated diagnosis, and was successfully completed on four out of five sample, as one sample did not meet the minimum tumor content cutoff of 50%. Methylation profiling provided an integrated diagnoses with variable results including: affirmed the diagnosis of neurocytoma (1/4), altered the diagnosis of neurocytoma (2/4), and neither affirmed or altered diagnosis (1/4). Calibrated scores ranged from 0.766 to 0.999 for samples that were ultimately able to be classified. A methylation clustering analysis t-distributed stochastic neighbor embedding (tSNE) plot is depicted in Fig. 3. Case 1 demonstrated methylation profiling consistent with central neurocytoma thus providing an integrated diagnosis of central neurocytoma with a calibrated score of 0.999— methylation profiling is not yet able to differentiate between atypical and typical neurocytomas. Case 2 did not demonstrate a match on methylation profiling, and in the context of known abnormal molecular drivers provided an integrated diagnosis of a neuroepithelial tumor with *EWSR1-ATF* fusion. Case 3 had an inadequate sample that did not meet the 50% tumor content cutoff for analysis, owing to the largely cystic nature of the tumor. Cases 4 and 5 demonstrated methylation profiling consistent with gangliogliomas with calibrated scores of 0.927 and 0.766 respectively, which differed from the initial histologic diagnosis of extraventricular neurocytomas.

All patients underwent surgical resection (gross-total 2/5, near-total 2/5, sub-total 1/5). Recurrence occurred in four children with two having multiple recurrences. Recurrences were treated with surgery (4/4), adjuvant radiation (2/4), and chemoradiation (1/4). Mean time to follow up was 77 months (range 44–112 months). The mean progression free survival and overall survival were 24 months (range 6 to 52 months) and 100% respectively.

## Discussion

Neurocytomas in the pediatric population have limited representation in the literature due to their low incidence [18, 19]. We provide a diverse five patient pediatric case series that represents histologically diagnosed central and extraventricular neurocytomas, typical and atypical neurocytomas, and recurrent neurocytomas. As neurocytomas generally exhibit benign characteristics and respond well to surgical intervention alone, all patients underwent initial surgical resection without adjuvant chemoradiation. Initial disease recurrence was managed by surgical intervention alone or with subsequent radiation therapy. Despite initial surgical interventions, four of the five patients demonstrated recurrent disease which differed markedly from a single institution long-term follow up of adult neurocytomas in which two thirds of patients had durable progression free survival after surgical resection alone [22]. This higher-than-expected disease recurrence may have been due to discordance between the histologic diagnosis and integrated diagnosis of these CNS tumors. Notably, despite a higher-than-expected recurrence rate, the patients in this series have had a 100% survival rate at time of last follow up (range 44–112 months).

This series demonstrates the wide range of radiological characteristics, tumor locations, and sizes that can be seen in the initial presentation of histologically diagnosed neurocytoma. One patient with nausea, vomiting, and visual disturbances was found to have central neurocytoma with secondary hydrocephalus on their MRI. Both patients with histologically diagnosed extraventricular neurocytoma had presenting symptoms of seizures and were initially managed with consideration for epilepsy surgery prior to their eventual tumor identification.

Histologic classification of tumors prior to molecular testing depends largely on proliferation architecture, cytologic form, nuclear details, and background features, which must be taken in the context of the broader clinical and radiologic differential. Diagnosing neurocytomas is often a challenging endeavor and is particularly difficult based on histology alone. Histologically, our tumor samples generally demonstrated a uniformly distributed, moderately to highly cellular proliferation, with round monotonous to moderately pleomorphic nuclei with stippled chromatin and eosinophilic cytoplasm (Fig. 2). The heterogeneity of these tumors became more apparent upon immunohistochemistry staining (Table 1), with two tumors (cases 1 and 2) demonstrating high rates of MIB-1 staining for Ki-67 antigen consistent with a diagnosis of atypical neurocytomas. There was recurrence of disease in both patients identified as having atypical features, with one patient having multiple recurrences, which is consistent with the higher rates of disease recurrence reported for atypical neurocytomas in the literature. Several histologic and molecular features of the tumor identified in case 2 raised question of other tumor diagnoses, particularly an intra-axial mesenchymal tumor of the CNS owing to the EWSR-ATF1 gene fusion. Ultimately, this diagnosis was lowered on the differential when external epithelial membrane antigen testing (EMA) was found to be negative and internal testing was found to only have scattered light positivity.

Next generation sequencing has proven as a useful tool in identifying the molecular drivers of individual tumors, and chromosomal microarray has been used to identify copy number variants that a tumor has accumulated in its course—but even with these molecular tools the diagnosis may remain elusive. In our cohort, next generation sequencing identified an *EWSR-ATF1* gene fusion and *MUTYH* mutation in one tumor (case 2), variants of uncertain significance in two tumors (cases 1 and 5), and no clinically significant finding in two tumors (cases 3 and 4). Clinically, case 2 had multiple recurrences and was more refractory to treatment than typical neurocytomas which may be due to its unique molecular drivers. Notably, there were no cancer predisposition germline mutations identified on whole genome sequencing of this patient, no family history of cancer, and no report of polyps identified in siblings to suggest a somatic type *MUTYH* variant. Case 3 highlights the utility of negative next generation sequencing, as histologically a septal dysembryoplastic neuroepithelial tumor (DNET) and myxoid glioneural tumor (GNT) were considered. As NGS was negative for *PDGFRA* mutations including p.k385, myxoid GNT was lowered on the differential [23]. Methylation analysis would have proved helpful in further narrowing the differential on this tumor, however this analysis could not be performed as the sample did not meet the minimum tumor content cutoff. Clinically, case 4 had multiple recurrences requiring repeated surgical interventions which is not typical for the expected clinical course for either neurocytoma or ganglioglioma.

Chromosomal microarray analysis generally does not demonstrate significant copy number abnormalities in neurocytomas, which is relatively consistent with the findings in our first three cases. The tumors ultimately classified as gangliogliomas had chromosomal microarrays with more broad copy number gains, thought to be less consistent with their histologic diagnosis of neurocytoma. Interestingly, gangliogliomas are typically also not associated with the significant broad copy number changes seen in cases 4 and 5, however they may rarely demonstrate gains in chromosome 7 and X, as seen in both cases. Diffuse glioneuronal tumor with oligodendroglioma-like features and nuclear clusters (DGONC) was additionally considered on the differential for case 5 due to its relatively low calibrated score for ganglioglioma on v11b6 methylation analysis, however the monosomy 14 more typically seen in DGONC was not seen on chromosomal microarray [24] and the tumor did not cluster with DGONC on v12 of the methylation classifier (publicly available, unpublished).

Methylation profiling has emerged as a powerful tool in the field of neuro-oncology, particularly in identifying new medulloblastoma subgroups that can aid in prognosis and management [25–29]. Though this diagnostic tool is not readily available for widespread clinical use, its strength in guiding integrated diagnoses and identifying new molecular drivers of disease suggests that it may have an emerging role in the clinical practice of neuro-oncology [29]. Methylation profiling affirmed our diagnosis of a central neurocytoma (calibrated score 0.999) in one case (case 1) and identified two cases of histologically diagnosed neurocytomas that clustered more closely with gangliogliomas, providing an alternative integrated diagnosis (calibrated scores 0.766—0.927). It should be noted that although methylation profiling was able to confidently classify the histologically diagnosed central neurocytoma in case 1 with a calibrated score of 0.999, this technique is not yet able to distinguish between typical and atypical neurocytomas. This series is the first to date to utilize methylation profiling results to provide an integrated diagnosis on histologically identified neurocytomas and highlights the diagnostic complexity of this rare brain tumor in the pediatric population. As both cases initially histologically diagnosed as extraventricular neurocytomas clustered with gangliogliomas on methylation profiling, this study highlights extraventricular location as a feature of histologically diagnosed neurocytomas that may benefit from increased diagnostic accuracy through this emerging molecular diagnostic technique.

This series is limited by its small sample size and lack of exclusion criteria, owing to the rarity of histologically diagnosed neurocytomas in the pediatric population. While this study demonstrates the potential future clinical utility of methylation profiling in providing an integrated diagnosis for difficult to diagnose CNS tumors, it also highlights the current limitations of available histologic and radiographic tools in distinguishing biologically similar rare CNS tumors. Two of four analyzed tumors in this study had methylation profiles that differed from their initial histologic diagnosis (calibrated scores 0.766–0.927), though it is challenging to interpret the significance of this finding as there are is no historical comparison for use of this novel technique in the integrated diagnosis of histologically diagnosed neurocytoma. Overall, this study highlights that neurocytomas warrant further investigation with particular attention to the role for next generation sequencing and methylation profiling in providing early and precise integrated diagnoses.

## Conclusion

Central and extraventricular neurocytomas are a rare collection of tumors that warrant further investigation. Next generation sequencing and methylation profiling are evolving techniques that may aid in providing early and precise integrated diagnoses to guide treatment strategies. Our series adds to the clinical and molecular complexity of central and extraventricular neurocytomas as methylation profiling may aid in the differentiation of neurocytoma from other difficult to diagnose tumors who share similar histologic features.
